# Targeting Longevity Gene *SLC13A5*: A Novel Approach to Prevent Age-Related Bone Fragility and Osteoporosis

**DOI:** 10.3390/metabo13121186

**Published:** 2023-12-06

**Authors:** Grit Zahn, Hannes A. Baukmann, Jasmine Wu, Jens Jordan, Andreas L. Birkenfeld, Naomi Dirckx, Marco F. Schmidt

**Affiliations:** 1 Eternygen GmbH, Westhafenstrasse 1, 13353 Berlin, Germany; 2 biotx.ai GmbH, Am Mühlenberg 11, 14476 Potsdam, Germanyms@biotx.ai (M.F.S.); 3 Department of Orthopaedics, School of Medicine, University of Maryland-Baltimore, Baltimore, MD 21201, USA; 4 German Aerospace Center (DLR), Institute of Aerospace Medicine, 51147 Cologne, Germany; jens.jordan@dlr.de; 5 Department of Diabetology Endocrinology and Nephrology, Internal Medicine IV, University Hospital Tübingen, Eberhard Karls University Tübingen, 72074 Tübingen, Germany; 6 German Center for Diabetes Research (DZD), Institute of Diabetes Research and Metabolic Diseases (IDM) of the Helmholtz Center Munich, Eberhard Karls University Tübingen, 72074 Tübingen, Germany; 7 Department of Diabetes, Life Sciences and Medicine, Cardiovascular Medicine and Sciences, Kings College London, London WC2R 2LS, UK

**Keywords:** mINDY, *SLC13A5*, citrate, citrate transporter, NaCT, osteoporosis, Mendelian randomization, drug development

## Abstract

Reduced expression of the plasma membrane citrate transporter *SLC13A5*, also known as INDY, has been linked to increased longevity and mitigated age-related cardiovascular and metabolic diseases. Citrate, a vital component of the tricarboxylic acid cycle, constitutes 1–5% of bone weight, binding to mineral apatite surfaces. Our previous research highlighted osteoblasts’ specialized metabolic pathway facilitated by *SLC13A5* regulating citrate uptake, production, and deposition within bones. Disrupting this pathway impairs bone mineralization in young mice. New Mendelian randomization analysis using UK Biobank data indicated that SNPs linked to reduced *SLC13A5* function lowered osteoporosis risk. Comparative studies of young (10 weeks) and middle-aged (52 weeks) osteocalcin-cre-driven osteoblast-specific *Slc13a5* knockout mice (*Slc13a5^cKO^*) showed a sexual dimorphism: while middle-aged females exhibited improved elasticity, middle-aged males demonstrated enhanced bone strength due to reduced *SLC13A5* function. These findings suggest reduced *SLC13A5* function could attenuate age-related bone fragility, advocating for *SLC13A5* inhibition as a potential osteoporosis treatment.

## 1. Introduction

The *SLC13A5* gene is the mammalian orthologue of the *I’m not dead yet (Indy)* gene in *Drosophila melanogaster* [1]. In mammals, the solute carrier family 13 member 5 (*SLC13A5*) protein, also called sodium-dependent citrate transporter (NaCT), is an electrogenic, sodium coupled tricarboxylate plasma membrane transporter with a preference for citrate [2]. *Slc13a5* knockout in mice protected high-fat-diet-fed and aged animals from adiposity and insulin resistance [3] and lowered blood pressure through sympathetic inhibition [4]. Thus, *SLC13A5* is a promising therapeutic target for metabolic-associated fatty liver disease (MAFLD) and associated cardiometabolic diseases [5,6,7].

However, *SLC13A5* also affects bone and tooth development as homozygous *Slc13a5*-deficient mice exhibited significant effects on bone and teeth mineralization [8,9]. In contrast, heterozygous knockout mice were not affected. In human beings, homozygous or compound heterozygous loss-of-function mutations in *SLC13A5* cause an autosomal-recessive disease with neonatal epilepsy, developmental delay, and teeth hypoplasia called EIEE25/DEE25 (early infantile epileptic encephalopathy-25/developmental epileptic encephalopathy-25) [10,11]. Remarkably, there are many significant differences between mice and human *SLC13A5* transporter biology such as gene expression, transport kinetic, and substrate specificity [2,12,13,14].

The *SLC13A5* substrate citrate is essential for the formation, structuring, and stabilization of apatite crystals in bone, as citrate comprises ~1.6% of the bone content, and about 80% of the total body citrate resides in bone [15,16]. Furthermore, *SLC13A5* is expressed in osteoblasts [9,17] and affects osteogenic differentiation of human mesenchymal stem cells into osteoblasts [17]. Osteoblasts take up citrate from circulation through *SLC13A5* [9] but can also produce their own citrate for bone formation in the TCA [16], and several dietary supplement studies showed the beneficial effects of a citrate supplementation diet on osteoporosis [18]. Overall, there is strong evidence that *SLC13A5* may affect bone health in human [8,9]. However, due to the substantial species differences between mouse and human, translational studies are urgently needed to understand the role of *SLC13A5* in human bone development and diseases.

Causal inference tools such as Mendelian randomization [19] (MR) or the recently published SynTrial workflow [20] are robust and accessible tools to examine the causal relationship between an exposure variable and an outcome from GWAS summary statistics as well as from biobank data at the individual level. Specifically, MR leverages randomly allocated genetic variants as instrumental variables for studying the effect of varying an exposure. The random allocation of genetic variants at conception means that this paradigm is less vulnerable to the environmental confounding and reverse causation bias that can hinder causal inference in traditional epidemiological study designs. A more recent extension of MR allows it to be applied to study the effects of drug target perturbation [21,22].

Our very recent study using MR for *SLC13A5* already identified 13 uncorrelated SNPs as instruments for *SLC13A5* inhibition and found associations between genetically proxied *SLC13A5* inhibition and improved kidney function as well as with higher plasma calcium levels and lower fasting glucose [23]. We here used MR and those 13 SNPs as an instrument to investigate the role of *SLC13A5* in human bone health. Furthermore, in order to establish a connection between the bone-related data obtained from our current and previously published studies conducted on young *Slc13a5*-deficient mice and our human genetic analysis, we conducted further investigations on bones obtained from middle-aged mice. These middle-aged mice are likely to be more representative of the population found in human genomic databases.

## 2. Materials and Methods

### 2.1. Mendelian Randomization

Mendelian randomization (MR) is a powerful method used in epidemiology and genetics leveraging genetic variants to act as natural experiments and allowing scientists to explore causal relationships between exposures and outcomes, providing valuable insights into disease mechanisms and potential therapeutic targets [21,22].

Summary statistics for clinically diagnosed and self-reported osteoporosis were produced by The Neale Lab (http://www.nealelab.is/uk-biobank/, accessed on 24 October 2022) following their GWAS analysis (version 3) of over 7000 phenotypes using samples from 361,194 individuals. Summary statistics for citrate were obtained from the MRC IEU OpenGWAS (https://www.biorxiv.org/content/10.1101/2020.08.10.244293v1.abstract, accessed on 5 August 2022).

For genome-wide Mendelian randomization experiments, a *p*-value threshold of 5 × 10^−8^ was applied to the exposure SNPs. Independent SNPs were identified by performing linkage disequilibrium (LD) clumping to ensure that genetic instruments were minimally correlated (r^2^ < 0.001) based on the European reference panel from the 1000 Genomes Project (https://www.internationalgenome.org/home, accessed on 8 May 2020) using the ld_clump() function of the R package ieugwasr (https://github.com/MRCIEU/ieugwasr, published on 8 May 2020), which provides a wrapper around PLINK [24]. For drug-target MR experiments, the 13 significant and independent SNPs were used as instruments [23].

The MR-Rücker framework [25] was used to decide whether IVW or MR-Egger is best supported by the data. Otherwise, the inverse-variance weighted (IVW method) for two SNPs or the Wald ratio method (one SNP) were used. All methods were implemented in the TwoSampleMR package [26].

### 2.2. Causal Biomarker Analysis

Complementary to MR analysis, we performed a causal biomarker analysis using synthetic clinical trial analysis, leveraging the rapidly expanding medical and genetic databases, computer modeling and simulation to replicate the processes of traditional clinical trials to get novel insights into disease mechanisms and potential therapeutic targets. Causal biomarker analysis, SynTrial, is described in detail in Baukmann et al. [20].

Using the rich information made available by the UK Biobank project [27], we defined osteoporosis cases as individuals that had reported ICD-10 M80 (“Osteoporosis with pathological fracture”), M81 (“Osteoporosis without pathological fracture”), or self-reported osteoporosis. Cases and controls were filtered for European ancestry (“White”, “British”, “Irish”, and “Any other white background”), and individuals with missing age and sex information were discarded. Controls were then matched to the same number of cases based on age and sex.

### 2.3. Animals

The *Slc13a5* floxed mice, in C57BL/6 background, were generously provided by Dr. Rafael De Cabo (NIA, Baltimore). In the skeletal-specific knockout model, the *Slc13a5* gene was recombined in osteoblasts (from embryonic day 17 onward) by crossing osteocalcin *(Oc)-Cre* [28] mice with *Slc13a5* floxed mice. Breeding pairs were set up between male *Oc-Cre^+/−^;Slc13a5^lox/lox^* and female *Oc-Cre^−/−^;Slc13a5^lox/lox^* to obtain progeny including *Slc13a5* conditional knockout (cKO) (*Oc-Cre^+/−^;Slc13a5^lox/lox^* and further referred to as *Slc13a5^cKO^*) mice and littermate controls (*Oc-Cre^−/−^;Slc13a5^lox/lox^*).

Genotyping was performed on ear punches using the following primers for *Slc13a5* KO allele: NADC5 loxF3 *GACTTAGCACAGCAGGTACT*, NADC5 frtR2 *GATCACTGTGATCTGGCCTA*, and for WT allele: NADC5 loxF3 *GACTTAGCACAGCAGGTACT*, NADC5 loxR *TTACCAACCACCTCGCTAGT*; Cre FW *GAA CCT GAT GGA CAT GTT CAG G*, Cre RV *AGT GCG TTC GAA CGC TAG AGC CTG T*.

Animals were euthanized by isoflurane inhalation and subsequent cervical dislocation. All breeding and experiments were performed according to Institutional Animal Care and Use Committee (IACUC) of the Johns Hopkins University and University of Maryland, Baltimore, guidelines and approved protocols.

### 2.4. Serum Analysis

Serum citrate (MAK333) and calcium (MAK022) levels were measured using a commercially available colorimetric assay and according to manufacturer’s protocol (Millipore Sigma, Saint Louis, MO, USA).

### 2.5. High-Resolution Micro-Computed Tomographic Imaging (Micro-CT)

An ex vivo micro-computed tomography (micro-CT) imaging system (Skyscan 1172 CT, Bruker, Kontich, Belgium) with corresponding Skyscan software v1.5.28 was used for mineral tissue analysis with 63 kV, 153 μA, 10 μm voxel size resolution and a 0.5 mm Al filter as scanning parameters. Samples were reconstructed using the Nrecon software (Bruker v2.0) with 30% beam hardening correction, ring artifact correction of 5, and CS to Image conversion of 0–0.09 for long bones. 3D reconstructed images were generated using CTvox software (Bruker v3.3.1). Ex vivo micro-CT analysis of the femur included both trabecular and cortical bone analyses and was performed using CTAn (Bruker v1.20.3.0). For trabecular bone quantification, a region of interest containing 100 slices (1 mm) was chosen, 25 slices (250 μm) below the growth plate. For cortical analysis, a region of interest was chosen containing 50 slices (500 μm) from the midpoint of the femur, 250 μm towards the proximal end and 250 μm towards the distal end. Bone volume/tissue volume (BV/TV, %), trabecular number (Tb. N, 1/mm), trabecular separation (Tb. Sp, mm), trabecular thickness (Tb. Th, mm), and cortical thickness (Cort. Th, mm) were determined using CTAn software (Bruker v1.20.3.0). Bone Mineral Density (BMD) and Tissue Mineral Density (TMD) were measured using the Bruker calibration phantoms of 0.25 and 0.75 gHA/cm^3^ and the ROIs generated for trabecular and cortical analysis, respectively.

### 2.6. Whole Bone Biomechanical Testing

To analyze the biomechanical properties of the femur, a low-force mechanical testing system (TA ElectroForce 3200 Series 3-Point Bending device, New Castle, DE, USA) and mat-lab software (version R2023b) was used. At dissection, the femur was harvested and frozen in −20 °C wrapped in a PBS-soaked gauze to keep it moist. The day before the test, specimens were thawed to room temperature in PBS, the mechanical properties of the femoral midshaft were assessed by 3-point bending by applying a flexion moment in the anterior-posterior plane with a span length of 7 mm. Preload was applied to the bones before testing to “train” the tissues. The force was applied at a constant displacement rate (0.05 mm/s) for 100 s, generating 2000 data points. The obtained force displacement data were used to determine yield load, failure load, stiffness, work-to-failure, and post-yield displacement. The material properties (Young’s modulus, stress, strain, and modulus of toughness) were obtained by adjusting the force displacement data for the appropriate femoral mid-shaft area moment of inertia, as measured by micro-CT.

## 3. Results

### 3.1. Mendelian Randomization

Citrate is the main substrate for *SLC13A5* and is proposed to affect overall citrate homeostasis. Gill et al. [23] described genetic variants at the *SLC13A5* locus which are robustly associated with circulating citrate levels at genome-wide significance, and which served as plausible genetic instruments for studying its effects in our study. We applied these 13 uncorrelated variants as instruments in drug target MR analyses.

We conducted two MR approaches: first, a genome-wide approach using all genome-wide significant genetic variants, and then a drug target approach in which we only used SNPs in the *SLC13A5* locus. In both cases, we utilized summary statistics on plasma citrate levels from Nightingale Health as the exposure and summary statistics on osteoporosis diagnosed by clinicians as well as on self-reported osteoporosis, both provided by the UK Biobank, as outcome.

**Figure 1:**
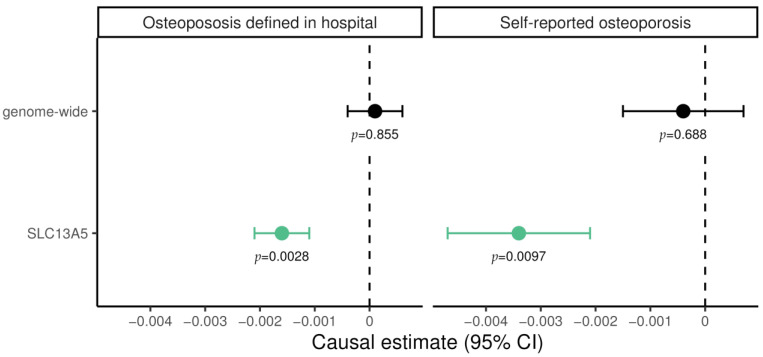
Mendelian randomization estimate for the causal effect of citrate on hospital-diagnosed and self-reported osteoporosis, restricted to variants in the *SLC13A5* gene and genome-wide, respectively. Causal effects were estimated using the inverse-variance weighted method. Green data points represent significant effects without pleiotropy. Estimates are scaled per 1 standard deviation (SD) increase in plasma citrate, i.e., every 1-SD higher genetically proxied plasma citrate through *SLC13A5* inhibition was associated with a 0.0034 lower log odds ratio for self-reported osteoporosis.

For genome-wide MR experiments, plasma citrate levels showed no evidence supporting causal effect on osteoporosis, neither on diagnosed (0.0001; 95% CI −0.0004 to 0.0006; *p* = 0.855) nor self-reported cases (−0.0004; 95% CI −0.0017 to −0.0007; *p* = 0.688; Figure 1). In contrast, for genetically predicted *SLC13A5* inhibition a significant lower risk of osteoporosis emerged both for diagnosed (−0.0016; 95% CI −0.0021 to −0.0011; *p* = 2.3 × 10^−3^) and for self-reported cases (−0.0034; 95% CI −0.0047 to −0.0021; *p* = 9.7 × 10^−3^). These results suggest a protective effect of *SLC13A5* inhibition on osteoporosis. The finding was not attributable to altered citrate levels more generally, as shown in genome-wide MR (Figure 1).

### 3.2. Causal Biomarker Analysis

Complementary to MR analysis, we performed a causal biomarker analysis using a previously developed synthetic clinical trial (SynTrial) workflow [20] to osteoporosis (Figure S1). Using UK Biobank data, we identified 9828 individuals with osteoporosis and used regression modelling to investigate the effect of 321 candidate predictive traits. A total of 89 traits significantly predicted osteoporosis with a Bonferroni-corrected significance threshold of *p* < ɑ/n = 0.05/321 (Table S1). After removing collinear traits, we applied drop-one analysis to compare all possible models that can be constructed by dropping a single model term and evaluating its impact on the regression model. The analysis revealed that three traits explain unique variance in osteoporosis status to a Bonferroni-corrected significance threshold of *p* < ɑ/n = 0.05/14 (Table S2). Propensity score analysis is a technique for estimating the treatment effects on an outcome independent of covariates. We employed propensity score stratification using the propensity function of Imai and van Dyk [29] to estimate treatment effects on osteoporosis independent of age, sex, BMI, and the other two traits, respectively. Heel BMD (estimate = −5.2377, *p* < 2.314 × 10^−177^), vitamin D (estimate = 0.0127, *p* < 2.323 × 10^−69^) and neutrophil/lymphocyte ratio (estimate = 0.1446, *p* < 2.546 × 10^−30^) were causal traits (Table 1). As expected, Heel BMD is a highly significant causal protective factor. Remarkably, like in MR analysis, there is no significant impact of citrate in general in causal biomarker analysis (Table S2).

### 3.3. Age-Induced Changes in Bone Morphology and Mechanical Properties in Skeletal-Specific Slc13a5^cKO^ Female and Male Mice

In a recent study by Dirckx et al., global or osteoblast-specific *Slc13a5* deletion caused increased mineral citrate levels and elicited mineralization defects resulting in reduced femur cortical thickness with increased fragility [9], which was in contrast to the genetic data showing protective effects on osteoporosis. However, these results were obtained in young mice (6 and 10 weeks old, respectively) that had not yet reached their peak bone mass. To elucidate age-related effects of *Slc13a5* deletion on bone, we phenotyped one-year-old male and female skeletal-specific knockout mice using the osteocalcin-cre driver strain, further referred to as *Slc13a5^cKO^* (Figure S2A for efficient recombination of *Slc13a5* in full bone RNA extracts) and compared them to control littermates. Although 1-year-old mice are only considered middle-aged, we did observe significant age-related bone loss in our model at this age (Figure S2B).

**Figure 2:**
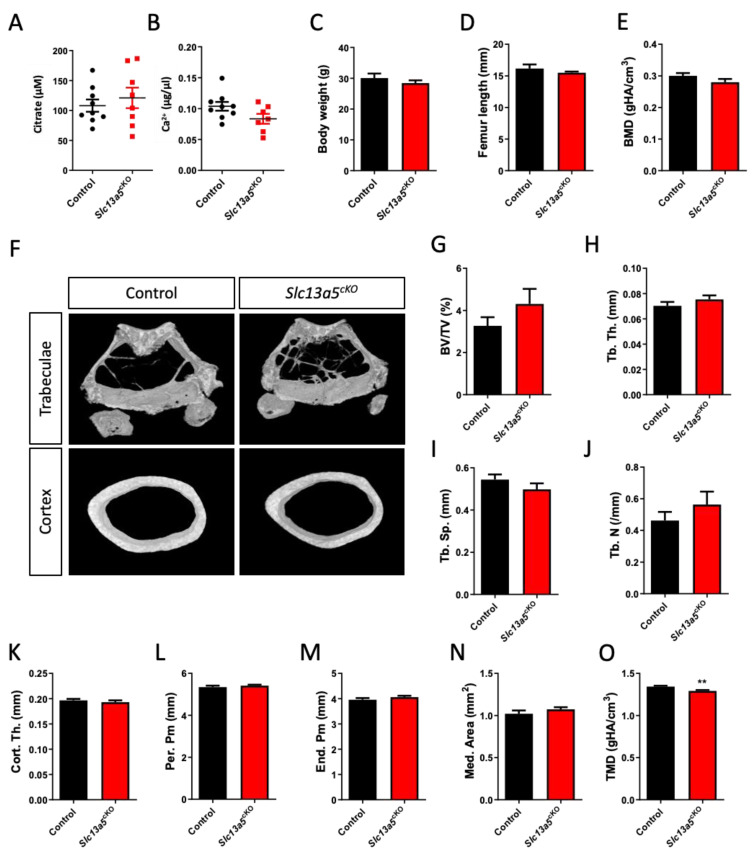
Phenotypical analysis of one-year-old female *Slc13a5^cKO^* mice. (**A**) Serum citrate levels (n = 8–9). (**B**) Serum Ca^2+^ levels (n = 7–9). (**C**) Body weight (n = 8–9). (**D**) Femur length (n = 8). (**E**–**O**) Micro-CT analysis of the femur in one-year-old control and *Slc13a5^cKO^* mice (n = 8–9) showing Bone Mineral Density (BMD, (**E**)), representative 3D micro-CT images from the analyzed trabecular and cortical bone area (**F**), Bone Volume/Tissue Volume (BV/TV%, (**G**)), Trabecular Thickness (Tb. Th., (**H**)), Trabecular Separation (Tb. Sp., (**I**)), Trabecular Number (Tb. N., (**J**)), Cortical Thickness (Cort. Th., (**K**)), Periosteal Perimeter (Per. Pm, (**L**)), Endosteal Perimeter (End. Pm., (**M**)), Medullary Area (Med. Area, (**N**)) and Tissue Mineral Density (TMD, (**O**)). All graphs represent mean ± SEM; Student’s *t*-test versus control, ** *p* < 0.01.

**Figure 3:**
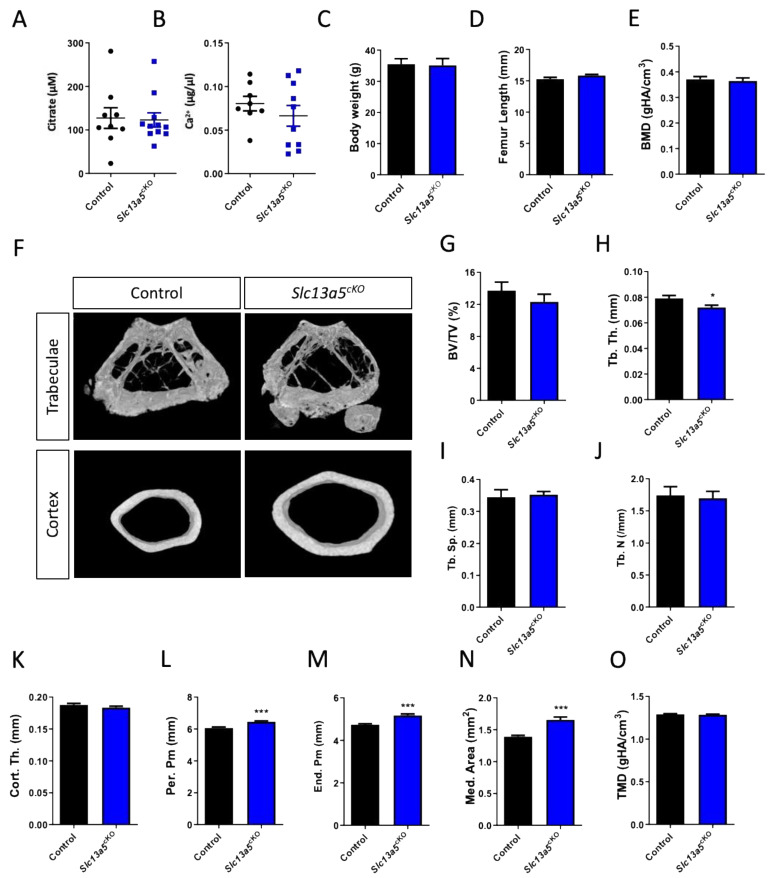
Phenotypical analysis of one-year-old male *Slc13a5^cKO^* mice. (**A**) Serum citrate levels (n = 9–11). (**B**) Serum Ca^2+^ levels (n = 8–10). (**C**) Body weight (n = 8). (**D**) Femur length (n = 9–11). (**E**–**O**) Micro-CT analysis of the femur in one-year-old control and *Slc13a5^cKO^* mice (n = 9–11) showing Bone Mineral Density (BMD, (**E**)), representative 3D micro-CT images from the analyzed trabecular and cortical bone area (**F**), Bone Volume/Tissue Volume (BV/TV%, (**G**)), Trabecular Thickness (Tb. Th., (**H**)), Trabecular Separation (Tb. Sp., (**I**)), Trabecular Number (Tb. N., (**J**)), Cortical Thickness (Cort. Th., (**K**)), Periosteal Perimeter (Per. Pm, (**L**)), Endosteal Perimeter (End. Pm., (**M**)), Medullary Area (Med. Area, (**N**)) and Tissue Mineral Density (TMD, (**O**)). All graphs represent mean ± SEM; Student’s *t*-test versus control, * *p* < 0.05, *** *p* < 0.001.

In both middle-aged female (Figure 2A,B) and male (Figure 3A,B) mice, we did not observe changes in serum citrate and calcium levels between controls and mutants. In both female (Figure 2C,D) and male mice (Figure 3C,D), age-related weight gain and adult growth was unaffected, as bodyweight and femur length did not differ between control and *Slc13a5^cKO^* mice. In female mice, detailed micro-CT analysis revealed that trabecular bone mass and bone mineral density (BMD) did not differ between control and *Slc13a5^cKO^* mice (Figure 2E–J), and was in line with what was observed in 10-week-old female mice [9], though a trend towards increased bone volume/tissue volume was observed in middle-aged *Slc13a5^cKO^* females (Figure 2F,G). Interestingly, cortical thickness, which was reduced by 20% in 10-week-old female *Slc13a5^cKO^* mice [9], was normal in one-year-old female *Slc13a5^cKO^* mice (Figure 2K) while the width of bone and medullary space remained unchanged (Figure 2L–N). However, similar to 10-week-old female mice [9], the cortical TMD was significantly reduced in one-year-old female mice (Figure 2O).

In one-year-old male mice, we did not observe differences in trabecular BMD or bone mass (Figure 3E–J), despite a reduction in trabecular thickness (Figure 3H) between control and *Slc13a5^cKO^* mice similar to 10-week-old male mice (Figure S3A–E). Cortical thickness was similar in both control and *Slc13a5^cKO^* mice in the young and middle-aged group (Figure 3K and Figure S3F), but femur width was significantly larger in middle-aged *Slc13a5^cKO^* mice compared to younger ones and controls (Figure 3L–N and Figure S3G–I). In young animals, we observed significant reductions in TMD (SI Figure S3J), which was normalized when the mice were middle-aged (Figure 3O).

Since our previous studies showed that altered citrate affects bone mass but more so bone quality [9], we next assessed bone mechanical properties by 3-point bending mechanical testing in young and middle-aged female and male mice.

In both, young (6 weeks, Table 2, left, and 10 weeks [9]) and middle-aged female *Slc13a5^cKO^* mice (Table 2, right), the femurs were more elastic (assessed using reduced Young’s modulus, reduced ultimate stress, and increased ultimate strain) than their control littermates. However, in young mice (6 weeks, Table 2, left, and 10 weeks [9]), increased elasticity was associated with a higher fragility as the ultimate moment (or load to fracture) was significantly reduced. Presumably, due to the substantially increased elasticity in the middle-aged female *Slc13a5^cKO^* mice (Table 2, right) and the reverted cortical thickness, the ultimate moment (or load to fracture) did not differ from their control littermates. In young male mice, we did not observe differences in mechanical strength (Table 3, left) while middle-aged male mice even showed a significantly increased ultimate moment (Table 3, right), which suggests that the bones were less fragile in the middle-aged mutants versus controls.

In summary, there are several structural differences between young and middle-aged *Slc13a5^cKO^* mice, and these differences also seem to be sex dependent. However, in both sexes, our data suggest that *Slc13a5* deficiency in middle-aged mice is rather beneficial compared to what was observed in young and growing mice with respect to their resistance to fracture. These findings align with the MR results (Figure 1).

## 4. Discussion

Using Mendelian randomization (MR) with human data and complementary animal studies, we gained novel mechanistic insights for citrate transporter *SLC13A5* and its role in bone diseases. The study suggests that reduced *SLC13A5* function may attenuate age-related bone fragility and underscores the potential of pharmacological inhibition of *SLC13A5* as an approach for the treatment of osteoporosis.

A recent study by Gill et al. [23] identified genetic variants at the *SLC13A5* locus which are robustly associated with circulating citrate. Therefore, citrate represents a biologically plausible biomarker to estimate *SLC13A5* activity, and these variants were used as genetic instruments for drug target MR analyses. Using drug target MR, we showed that genetically proxied *SLC13A5* inhibition is associated with a lower risk of both clinically diagnosed and self-reported osteoporosis in UK Biobank participants. The association was not attributable to altered citrate levels more generally, despite greater statistical power of this measurement. In accordance with the results of the genome-wide MR experiment, circulating citrate was not a causal factor for osteoporosis in the SynTrial. This observation may suggest that the effect on osteoporosis is mediated through *SLC13A5* and citrate uptake by the relevant cells independently of actions on plasma citrate levels, or that plasma citrate is a heterogeneous trait that is affected through several distinct pathways, and that at least some of these may be beneficial for bones. Similar observations in MR studies were described for the association of lipids and cancer [30,31]. Variants in the *HMGCR* locus were associated with breast cancer outcomes, but not with genome-wide variants for LDL-cholesterol, indicating there may be an off-target non-LDL-C-based mechanism regulated by HMGCR.

In our causal biomarker analysis using a SynTrial method, we have identified heel bone mineral density (Heel BMD), vitamin D, and neutrophil/lymphocyte ratio (NLR) as causal traits for osteoporosis. Previous MR studies showed that bone mineral density is a highly significant causal protective factor, is the gold standard for clinical osteoporosis assessment, and, therefore, serves as a positive control of our method [32,33]. In contrast, epidemiological as well as genetic data for vitamin D are conflicting, despite widespread prescription in osteoporosis prevention and treatment [33,34]. The discrepancies may result from heterogeneity of study populations and widely used vitamin D supplementation. Indeed, in the UK Biobank, 21.34% of osteoporosis cases were on vitamin D supplements, compared to 6.91% in control persons. The impact of NLR on osteoporosis found in our synthetic clinical trial confirms data from several prospective and cross-sectional clinical studies in osteoporosis patients [35,36,37]. However, no novel traits could be identified.

Several citrate dietary supplement studies in patients showed beneficial effects on osteoporosis [18] which was not supported by our genetic data analysis. In fact, our study did not reveal any beneficial effects of altered citrate. Possibly, the discrepancy results from differences between small, lifelong changes in genetically predicted citrate levels and large changes in citrate availability on supplements. Furthermore, other variants causing changes in citrate plasma level may have possible opposite or compensatory effects on bone metabolism. Furthermore, few studies showed no or only limited effects of citrate diet on bone turnover [18]. Finally, most clinical studies testing the impact of citrate diet on postmenopausal osteoporosis were performed in small cohorts and often combined with calcium and vitamin D supplementation [18]. Therefore, further well-controlled and larger clinical studies are needed to understand the impact of citrate diet on bone health.

The particularly high hepatic *SLC13A5* expression in humans (www.proteinatlas.org, accessed on 24 August 2023) suggests that the liver is an important organ for citrate elimination from circulation, and therefore, regulation of plasma citrate concentrations. The idea is supported by a previous metabolomics analysis in homozygous patients with loss-of-function mutations who showed 3-fold higher plasma citrate concentrations compared with healthy control persons [38]. In *Slc13a5*-deficient mice, plasma citrate was elevated in some but not in all studies by 10–100%, which may be explained by much lower hepatic *SLC13A5* expression in mice than in human beings [3,9,39]. Furthermore, modelling of plasma citrate flux into the liver based on pharmacokinetic data from healthy human subjects suggested that the liver is the major organ of citrate clearance from plasma in humans [40]. Other studies showed that hepatic clearance can be further increased under certain conditions such as surgery causing hypocitricemia [41]. However, the closely related transporters *SLC13A2* and *SLC13A3*, mainly expressed in the kidney, may also contribute to citrate clearance. In *Slc13a2* knockout mouse, urinary citrate excretion was increased with unchanged plasma citrate concentration [42]. A recently reported human *SLC13A3* variant with lower transporter function showed no effects on urinary citrate levels, but plasma citrate levels were not reported [43]. Furthermore, *SLC13A2* and *SLC13A3* were not associated with changes in plasma citrate concentration in AstraZeneca PheWAS Portal [44].

The protective effects of altered *SLC13A5* for osteoporosis proxied by genetic variants (SNPs) appear to contradict to previously published findings in *Slc13a5*-deficient mice which showed reduced bone mineral density and impaired bone mineralization leading to more fragile bones [8,9]. The discrepancy might be explained by biological differences between small, lifelong changes in carriers of genetic variants in the UK Biobank and complete loss of function in *Slc13a5*-deficient mice. Impaired bone health was only observed in homozygous knockout, whereas heterozygous animals were unaffected. Furthermore, the time point of observations may play a role as well. UK Biobank data are collected in adults of an average age of 56 years, while *Slc13a5*-deficient mice have previously been studied at a young age. Indeed, a previous study showed negative effects on bone mineralization at 13 weeks but not at week 32 [8,9]. Another mouse model of bone disease, osteogenesis imperfecta (OI), showed 2.5-fold increased *SLC13A5* expression levels in bone and abnormal mineralization [45] and may provide another hint for beneficial effects of *SLC13A5* inhibition on bone health. In line herewith, another recent publication showed that increased expression of *Slc13a5* caused a progeria-like phenotype in both male and female mice with reduced bone density [46].

Intriguingly, our studies on one-year-old skeletal-specific *Slc13a5*-deficient mice reveal similarities with what was predicted with inhibitory *SLC13A5* gene variants in humans. Although we only observed mild improvements regarding bone mass in middle-aged male and female *Slc13a5^cKO^* mice, changes at the ultrastructural level determining bone quality were more pronounced. Indeed, while young female *Slc13a5^cKO^* mice (6 weeks and 10 weeks) show reduced resistance to fracture, middle-aged female *Slc13a5^cKO^* mice appear to have changed their material properties and developed enhanced elasticity in their bones, leading to a resistance to fracture that is comparable to their wildtype littermates. Young male *Slc13a5^cKO^* mice had a similar bone strength to their control littermates, while middle-aged *Slc13a5^cKO^* mice had a significantly increased ultimate moment compared to controls. As such, *SLC13A5* appears to differentially affect young versus middle-aged and male versus female mice. The mechanisms behind the differential phenotype in one-year-old male and female *Slc13a5^cKO^* mice remain speculative and are presumably attributed to physiological [18] and endocrine differences [47] in males and females that accumulate with age and differentially affect both citrate homeostasis and bone quality in both sexes. However, despite the morphological differences, in both sexes, the mechanical properties become more favorable with age upon *Slc13a5* deletion and are, therefore, in agreement with our genetic data obtained in human subjects. We previously reported that young *Slc13a5*-deficient mice showed increased levels of mineral citrate which proved to be detrimental for bone mineralization and strength during growth [9]. However, it has been shown that osteoporosis was associated with reduced citrate levels in plasma and bone [48] in both human and mice. Therefore, we speculate that the increased citrate accumulation in bone minerals from *Slc13a5^cKO^* mice serves a more protective role against age-induced bone fragility in the one-year-old study group. It is noteworthy that these middle-aged *Slc13a5^cKO^* mice were mutant since birth. Follow up studies with inducible *Slc13a5* knockouts at an older age or administration of the *SLC13A5* inhibitor in osteoporotic mice will provide further valuable insights into the beneficial effects of and mechanisms behind *SLC13A5* inhibition in osteoporosis management.

Patients with loss of function mutations are not routinely tested for bone disease. Yet, investigations on non-neurologic health of patients with autosomal recessive *SLC13A5* Citrate Transporter (NaCT) Disorder reported tooth abnormalities caused by hypomineralized dentin and enamel [9] in most patients without overt skeletal disease (e.g., deformities or spontaneous fractures), except for a trend of slower growth [49]. Our study provides an impetus studying implication of *SLC13A5* loss of function in humans in more detail. An ongoing NHS study (NCT04681781) may provide further insights. However, significant species differences in target biology may result in different phenotypes in humans and mice with genetic *Slc13a5* deficiencies. In particular, differences in transport kinetics could lead to different outcomes associated with *Slc13a5*-deficient mice or mutations or other variants in human *Slc13a5* [13,50]. The high affinity/low capacity transporter in mice is completely saturated under physiological plasma citrate concentrations of approximately 150–200 μM and cannot respond to further increases in circulating citrate, whereas the human low affinity/high capacity transporter is not saturated, and therefore, can respond to changes in citrate levels [2,51]. Additionally, there are significant differences in gene expression level and tissue distribution. In humans, *SLC13A5* is mainly expressed in the liver at levels several orders of magnitude higher than in all other tissues (https://gtexportal.org/home/gene/SLC13A5, accessed on 24 August 2023) whereas in mice, the transporter is mainly expressed in incisors, bone, brain, and testis [9]. Data on expression in bone or bone cells in both species are limited; however, the available data indicate that bone expression of *SLC13A5* appears to be higher in mice than in human beings (https://genevisible.com/tissues/MM/Gene%20Symbol/Slc13a5, accessed on 24 August 2023), but it also changes during bone development [52]. We have recently shown that in vitro mouse osteoblasts expression of *SLC13A5* increases more than 20-fold over the course of osteoblast differentiation compared to only 3-fold in humans [9].

Overall, our data suggest that pharmacological *SLC13A5* inhibition could have utility in preventing or treating osteoporosis. Furthermore, epidemiological studies suggest a link between metabolic diseases, such as non-alcoholic fatty liver disease, and osteoporosis [53].

## 5. Conclusions

Overall, based on the presented data, pharmacological inhibition of *SLC13A5* function may be considered as a promising new approach to treat osteoporosis. Although several drugs are already approved for osteoporosis with significant improvements in the last few years, there is still a high medical need for well-tolerated and long-term-efficient drugs [54,55,56]. Interestingly, there are several epidemiological studies linking metabolic diseases in particular non-alcoholic fatty liver disease and osteoporosis [53,57]. Those studies showed consistently that the prevalence and risk of osteoporosis or osteoporotic fractures were significantly associated with NAFLD in men and women. As *SLC13A5* inhibitors are under development for fatty liver and NASH [7], synergies may be possible for patients with metabolic diseases and osteoporosis co-morbidity. However, further mechanistic studies are needed to better understand the impact of *SLC13A5* inhibition in human bone metabolism.

## Figures and Tables

**Table 1:** Results from Propensity Score Analysis (PSA), the last step of the Synthetic Clinical Trial workflow.

Trait	Estimate	Standard Error	*p* Value
Heel BMD	−5.2377	0.205	2.314 × 10^−177^
Vitamin D	0.0127	0.001	2.323 × 10^−69^
Neutrophil/ lymphocyte ratio	0.1446	0.018	2.546 × 10^−30^

**Table 2:** Table representing all 3-point bending mechanical testing parameters in 1-year-old female control and *Slc13a5^cKO^* mice (right, n = 8–9) compared to 6-week-old female control and *Slc13a5^cKO^* mice (left, n = 8–11). Parameters that are significantly increased or decreased are represented in orange and green, respectively, Student’s *t*-test versus control.

	6 Weeks Females					1 Year Females				
	Control		*Slc13a5^cKO^*		*p*-Value	Control		*Slc13a5^cKO^*		*p*-Value
	Mean	StDev	Mean	StDev		Mean	StDev	Mean	StDev	
Ultimate Moment (Nmm)	16.306	0.681	14.853	1.279	**0.010**	22.754	2.613	22.960	2.226	0.864
Bending Rigidity (Nmm^2^)	340.344	51.487	309.758	50.248	0.212	671.574	149.997	728.670	121.604	0.406
Ultimate Stress (MPa)	80.458	5.095	68.330	8.026	**0.003**	480.285	86.127	208.860	206.639	**0.003**
Young’s Modulus (MPa)	3023.970	574.458	2446.854	479.900	**0.035**	43,622.800	10,573.766	19,982.679	24,105.944	**0.017**
Ultimate Displacement (mm)	1.540	0.514	2.445	1.052	**0.051**	0.434	0.180	0.485	0.044	0.451
Pre-Yield Displacement (mm)	0.197	0.027	0.224	0.042	0.126	0.101	0.025	0.102	0.020	0.902
Post-Yield Displacement (mm)	1.345	0.533	2.221	1.041	**0.058**	0.334	0.166	0.383	0.048	0.433
Ultimate Strain (mm/mm)	0.220	0.078	0.349	0.146	**0.048**	0.034	0.015	0.065	0.019	**0.002**
Pre-Yield Strain (mm/mm)	0.028	0.004	0.032	0.006	0.127	0.008	0.002	0.014	0.006	**0.010**
Post-Yield Strain (mm/mm)	0.243	0.161	0.317	0.145	0.305	0.026	0.013	0.051	0.014	**0.002**
Ultimate Bending Energy (J)	10.567	3.991	11.761	3.127	0.474	6.323	1.805	5.122	0.785	0.103
Pre-Yield Energy (J)	1.069	0.214	1.133	0.303	0.618	0.585	0.213	0.588	0.215	0.978
Post-Yield Energy (J)	9.498	4.033	10.628	3.058	0.496	5.738	1.916	4.534	0.674	0.113
Toughness (J/mm^3^)	10.926	2.555	13.662	4.403	0.158	19.121	9.140	8.446	6.779	**0.016**
Pre-Yield Toughness (J/mm^3^)	1.283	0.342	1.317	0.429	0.857	1.678	0.660	0.934	0.770	**0.049**
Post-Yield Toughness (J/mm^3^)	9.664	2.622	12.345	4.209	0.153	17.443	9.362	7.512	6.051	**0.022**

**Table 3:** Table representing all 3-point bending mechanical testing parameters in 1-year-old male control and *Slc13a5^cKO^* mice (right, n = 9–11) compared to 10-week-old male control and *Slc13a5^cKO^* mice (left, n = 8–9). Parameters that are significantly increased or decreased are represented in orange and green, respectively, Student’s *t*-test versus control.

	10 Weeks Males					1 Year Males				
	Control		*Slc13a5^cKO^*		*p*-Value	Control		*Slc13a5^cKO^*		*p*-Value
	Mean	StDev	Mean	StDev		Mean	StDev	Mean	StDev	
Ultimate Moment (Nmm)	25.723	4.099	25.756	5.869	0.989	24.204	3.372	27.381	2.881	**0.035**
Bending Rigidity (Nmm^2^)	643.472	111.209	587.465	174.759	0.437	714.243	123.466	709.637	143.268	0.940
Ultimate Stress (MPa)	69.083	13.541	58.256	20.525	0.214	234.489	185.895	347.755	43.898	**0.065**
Young’s Modulus (MPa)	563.832	157.208	434.808	176.560	0.132	16,368.289	14,239.261	23,053.527	5815.719	0.171
Ultimate Displacement (mm)	1.256	0.604	1.553	0.662	0.349	0.689	0.367	0.688	0.422	0.995
Pre-Yield Displacement (mm)	0.124	0.032	0.118	0.048	0.760	0.108	0.017	0.134	0.047	0.137
Post-Yield Displacement (mm)	1.131	0.587	1.435	0.664	0.333	0.581	0.362	0.554	0.400	0.878
Ultimate Strain (mm/mm)	0.953	0.465	1.144	0.467	0.412	0.089	0.051	0.067	0.043	0.305
Pre-Yield Strain (mm/mm)	0.095	0.023	0.086	0.029	0.528	0.014	0.004	0.013	0.005	0.758
Post-Yield Strain (mm/mm)	0.859	0.452	1.058	0.478	0.392	0.076	0.049	0.054	0.040	0.293
Ultimate Bending Energy (J)	12.364	3.020	14.518	4.497	0.259	7.321	3.489	7.429	4.005	0.950
Pre-Yield Energy (J)	0.717	0.265	0.725	0.374	0.956	0.710	0.179	1.046	0.625	0.138
Post-Yield Energy (J)	11.647	2.995	13.792	4.654	0.271	6.611	3.421	6.383	3.686	0.889
Toughness (J/mm^3^)	45.566	18.311	43.979	22.559	0.875	12.918	12.023	16.019	8.771	0.513
Pre-Yield Toughness (J/mm^3^)	2.587	0.897	2.238	1.488	0.561	1.279	0.983	2.262	1.442	0.099
Post-Yield Toughness (J/mm^3^)	42.979	17.866	41.741	22.015	0.900	11.639	11.189	13.757	7.953	0.627

## Data Availability

Data are contained within the article and supplementary materials.

## Supplementary Materials

The following supporting information can be downloaded at: https://www.mdpi.com/article/10.3390/metabo13121186/s1, Figure S1. Workflow and results of the synthetic clinical trial on osteoporosis. Figure S2. Relative *Slc13a5* mRNA expression in full bones of control and *Slc13a5^cKO^* mice and Bone Volume/Tissue Volume (BV/TV)% in male and female control mice at 10 (young) and 52 weeks (middle-aged) of age. Figure S3. Micro-CT analysis of the femur in 10-week-old control and *Slc13a5^cKO^* male mice. Table S1. Results of the regression on osteoporosis. Table S2. The F statistic and their probabilities Pr(>F) values of traits determined in drop-one analysis.
