# Supplementary material for: Targeting Longevity Gene SLC13A5: A Novel Approach to Prevent Age-Related Bone Fragility and Osteoporosis
Source: Metabolites. 2023 Dec 6;13(12):1186. doi: 10.3390/metabo13121186 (PMC10744747; doi:10.3390/metabo13121186)
Supplement: Supplementary file 1 [file metabolites-13-01186-s001.zip › metabolites-2737950-supplementary.pdf]

Supplementary material

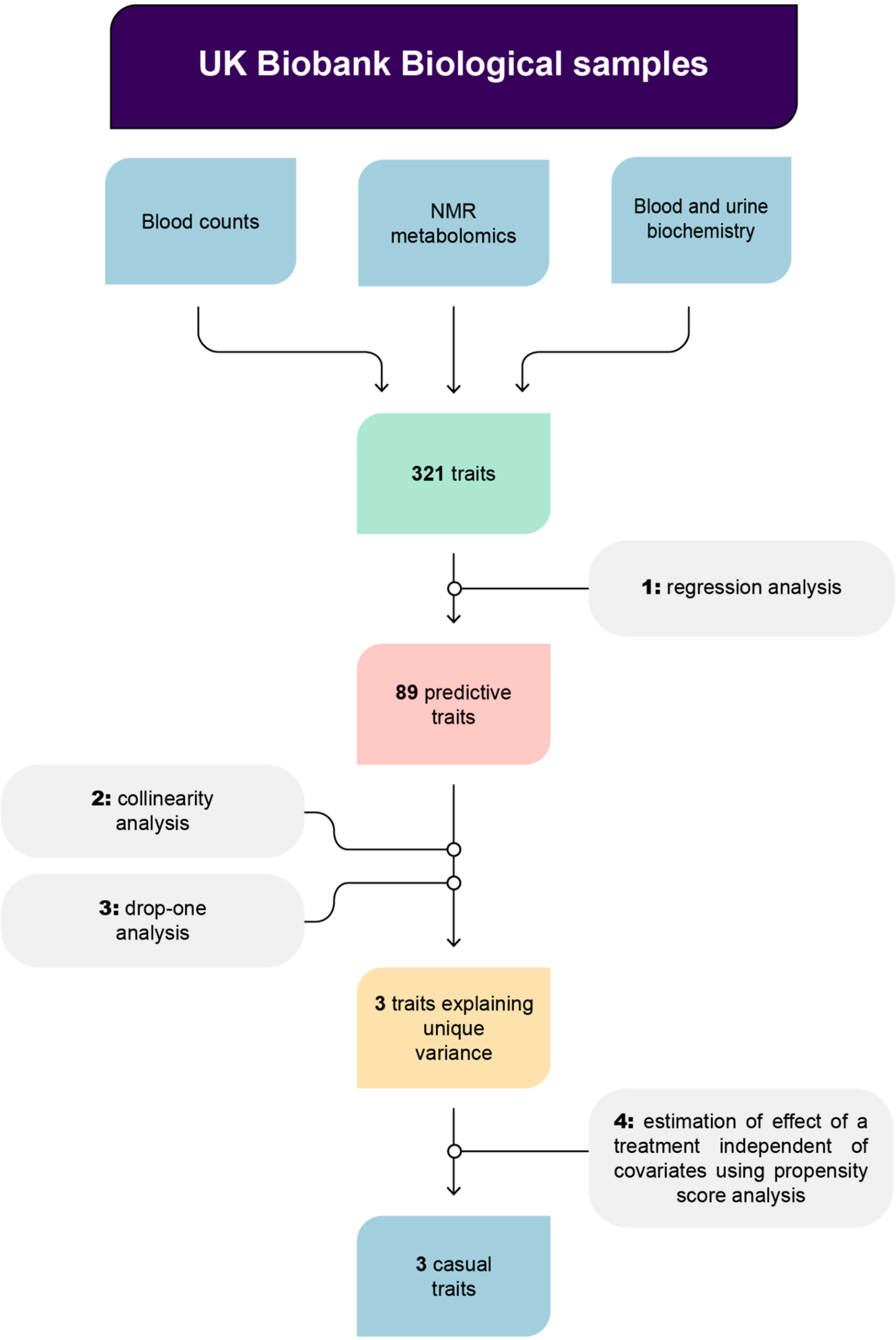

Figure S1. Workflow and results of the synthetic clinical trial on osteoporosis.

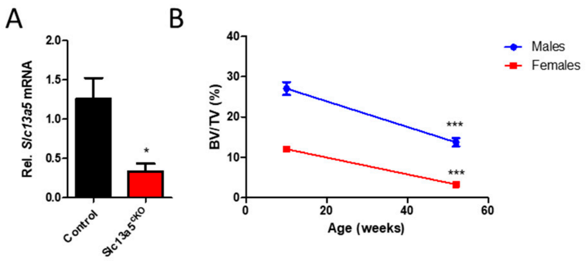

Figure S2. A) Relative *Slc13a5* mRNA expression in full bones of control and *Slc13a5*^{cKO} mice (n=4). B) Bone Volume/Tissue Volume (BV/TV) % in male and female control mice at 10 (young) and 52 weeks (middle-aged) of age (n=6-11). All graphs represent mean ± SEM; Student's t-test versus control (A) or young (B), **p*<0.05, ****p*<0.001.

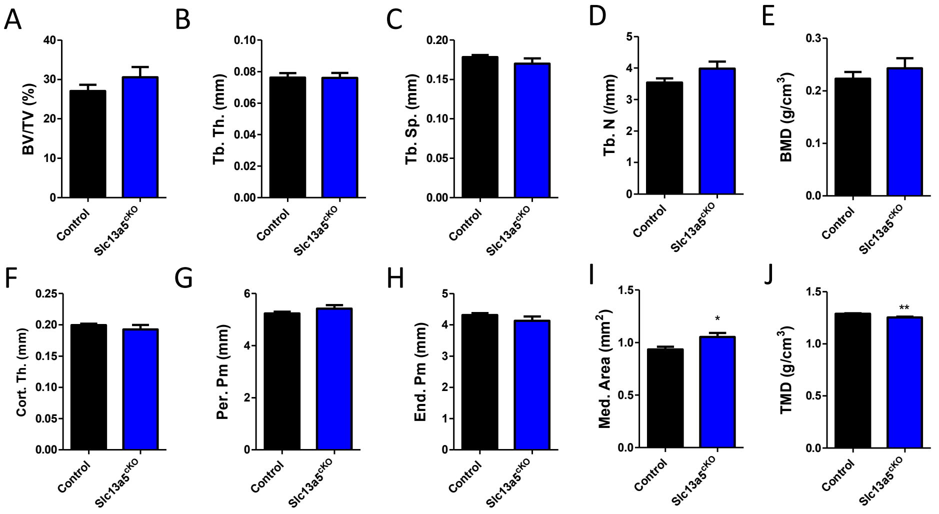

Figure S3. (A-H) Micro-CT analysis of the femur in 10-week-old control and *Slc13a5*^{cKO} male mice (n=8-11) showing Bone Volume/Tissue Volume (BV/TV%, A), Trabecular Thickness (Tb. Th., B), Trabecular Separation (Tb. Sp., C), Trabecular Number (Tb. N., D), Bone Mineral Density (BMD, E) Cortical Thickness (Cort. Th., F), Periosteal Perimeter (Per. Pm, G), Endosteal Perimeter (End. Pm., H), and Medullary Area (Med. Area, I), and Tissue Mineral Density. All graphs represent mean ± SEM; Student's t-test versus control, **p*<0.05, ***p*<0.01.

Table S1. Results of the regression on osteoporosis. Significance thresholds are indicated by asterisks, where three asterisks indicate *p*-values below 0.001/51, two indicate *p*-values below 0.01/51, and one asterisk indicates *p*-values below 0.05/51.

**Trait**	**Estimate**	**SE**	***p*-value**
**bmi**	-0.0591	0.0031	2.5332E-83
**high_light_scatter_reticulocyte_ct**	-7.6383	1.5321	6.1851E-07
**high_light_scatter_reticulocyte_perc**	-0.2015	0.0644	0.0018
**red_blood_cell_ct**	-0.4964	0.0385	4.0573E-38
**red_blood_cell_distribution_width**	0.1563	0.0152	6.6728E-25
**mean_reticulocyte_vol**	0.0178	0.0018	6.3137E-22
**immature_reticulocyte_fraction**	-0.5717	0.2372	0.0159
**reticulocyte_ct**	-1.9724	0.524	2E-04
**reticulocyte_perc**	-0.0438	0.0188	0.0197
**nucleated_red_blood_cell_ct**	0.7691	0.6059	0.2043
**nucleated_red_blood_cell_perc**	0.0673	0.0409	0.0999
**haemoblogin_concentration**	-0.1599	0.0134	1.1526E-32

**mean_corpuscular_volume**	0.0214	0.0032	4.2703E-11
**mean_corpuscular_haemoglobin**	0.0225	0.0075	0.0026
**mean_corpuscular_haemoglobin_concentration**	-0.0505	0.0134	2E-04
**haematocrit_perc**	-0.0474	0.0046	3.1836E-25
**platelet_ct**	9E-04	2E-04	1E-04
**platelet_distribution_width**	-0.138	0.0283	1.0439E-06
**platelet_crit**	0.4769	0.2831	0.0921
**mean_platelet_vol**	-0.063	0.0133	2.2162E-06
**white_blood_cell_ct**	0.033	0.0076	0
**basophill_ct**	0.912	0.2969	0.0021
**basophill_perc**	0.0393	0.0241	0.1028
**eosinophill_ct**	0.123	0.107	0.2504
**eosinophill_perc**	-0.0034	0.0081	0.6697
**neutrophill_ct**	0.0849	0.01	1.7377E-17
**neutrophill_perc**	0.0151	0.0017	2.5068E-19
**monocyte_ct**	0.1117	0.0657	0.089
**monocyte_perc**	7E-04	0.0053	0.8942
**lymphocyte_ct**	-0.0898	0.0203	0
**lymphocyte_perc**	-0.0195	0.0019	1.1704E-24
**mean_sphered_cell_vol**	0.0266	0.0027	9.7436E-23
**alanine_aminotransferase**	-0.0047	0.0011	0
**albumin**	-0.0326	0.0057	1.1261E-08
**alkaline_phosphate**	-0.0011	5E-04	0.015
**apolipoprotein_a**	0.1709	0.0548	0.0018
**apolipoprotein_b**	-0.501	0.0612	2.7207E-16
**aspartate_aminotransferase**	0.0034	0.0014	0.0168
**direct_bilirubin**	0.0195	0.0167	0.243
**total_bilirubin**	-0.0123	0.0037	8E-04
**c_reactive_protein**	0.0126	0.0029	0
**calcium**	0.4157	0.1485	0.0051
**cholesterol**	-0.0676	0.0125	6.6418E-08
**creatinine**	-6E-04	7E-04	0.3929
**cystatin_c**	0.508	0.078	7.4832E-11
**gamma_glutamytransferase**	0.0013	3E-04	0
**glucose**	-0.0274	0.0131	0.0362
**glycated_haemoglobin**	-0.0061	0.0023	0.0073
**hdl_cholesterol**	0.2275	0.0379	1.899E-09
**igf_1**	-0.0115	0.0026	9.2223E-06
**ldl_direct**	-0.1286	0.0165	5.9561E-15
**lipoprotein_a**	4E-04	3E-04	0.2802
**oestradiol**	-3E-04	1E-04	0.0805
**phosphate**	0.0874	0.0975	0.37
**rheumatoid_factor**	0.0043	0.0022	0.0481
**shbg**	0.0077	5E-04	2.177E-48
**testosterone**	0.0053	0.0034	0.1178
**total_protein**	-0.0109	0.0036	0.0026

**triglycerides**	-0.0982	0.0161	1.1269E-09
**urage**	-0.0023	2E-04	2.4821E-30
**urea**	-0.012	0.0096	0.2105
**vitamin_d**	0.0127	7E-04	7.2396E-72
**creatinine_urin**	0	2.8337E-06	8.0977E-06
**microalbumin_urin**	3E-04	2E-04	0.1666
**potassium_urin**	-0.0023	4E-04	2.5229E-07
**sodium_urin**	-0.0019	4E-04	2.1553E-07
**3_hydroxybutyrate**	0.923	0.4862	0.0577
**acetate**	1.8136	0.9394	0.0535
**acetoacetate**	6.3061	2.4014	0.0086
**acetone**	10.8044	5.2939	0.0413
**alanine**	-0.8108	0.3951	0.0402
**albumin_nmr**	-0.0266	0.0088	0.0026
**apolipoprotein_a1_nmr**	-0.0315	0.1193	0.7916
**apolipoprotein_b_nmr**	-0.5495	0.1443	1E-04
**apolipoprotein_b_over_apolipoprotein_a1_nmr**	-0.6113	0.1842	9E-04
**average_diameter_hdl_particles**	0.6111	0.1388	0
**average_diameter_ldl_particles**	0.3068	0.3469	0.3764
**average_diameter_vldl_particles**	-0.0843	0.0244	5E-04
**cholesterol_in_chylomicrons_and_extremely_large_vldl**	-1.9122	0.7168	0.0076
**cholesterol_in_idl**	-0.3713	0.1313	0.0047
**cholesterol_in_large_hdl**	0.4716	0.1648	0.0042
**cholesterol_in_large_ldl**	-0.4102	0.1017	1E-04
**cholesterol_in_large_vldl**	-2.5426	0.6264	0
**cholesterol_in_medium_hdl**	0.0149	0.2387	0.9503
**cholesterol_in_medium_ldl**	-1.2807	0.252	3.7137E-07
**cholesterol_in_medium_vldl**	-1.4664	0.4357	8E-04
**cholesterol_in_small_hdl**	-2.2138	0.4733	2.9106E-06
**cholesterol_in_small_ldl**	-3.0406	0.6681	5.3339E-06
**cholesterol_in_small_vldl**	-2.0692	0.557	2E-04
**cholesterol_in_very_large_hdl**	2.3338	0.7608	0.0022
**cholesterol_in_very_large_vldl**	-4.0678	1.0483	1E-04
**cholesterol_in_very_small_vldl**	-1.0505	0.5661	0.0635
**cholesterol_to_total_lipids_in_cylomicrons_and_extremely_large_vldl_percentage**	-0.0036	0.0026	0.1596
**cholesterol_to_total_lipids_in_idl_percentage**	-0.0211	0.01	0.0349
**cholesterol_to_total_lipids_in_large_hdl_percentage**	0.0121	0.006	0.0438
**cholesterol_to_total_lipids_in_large_ldl_percentage**	-0.0393	0.0178	0.0274
**cholesterol_to_total_lipids_in_large_vldl_percentage**	0.0134	0.0066	0.0409
**cholesterol_to_total_lipids_in_medium_hdl_percentage**	0.0146	0.0101	0.149
**cholesterol_to_total_lipids_in_medium_ldl_percentage**	-0.0593	0.0178	9E-04
**cholesterol_to_total_lipids_in_medium_vldl_percentage**	0.0067	0.0044	0.1266
**cholesterol_to_total_lipids_in_small_hdl_percentage**	-0.0198	0.0162	0.2229
**cholesterol_to_total_lipids_in_small_ldl_percentage**	-0.0672	0.0149	6.4803E-06
**cholesterol_to_total_lipids_in_small_vldl_percentage**	-3E-04	0.0063	0.9624

**cholesterol_to_total_lipids_in_very_large_hdl_percentage**	-0.0291	0.0078	2E-04
**cholesterol_to_total_lipids_in_very_large_vldl_percentage**	0.0071	0.0035	0.0404
**cholesterol_to_total_lipids_in_very_small_vldl_percentage**	-0.0024	0.0066	0.7164
**cholesteryl_esters_in_chylomicrons_and_extremely_large_vldl**	-3.8764	1.2996	0.0029
**cholesteryl_esters_in_hdl**	0.1151	0.1125	0.3064
**cholesteryl_esters_in_idl**	-0.5082	0.1766	0.004
**cholesteryl_esters_in_ldl**	-0.4126	0.0906	5.3097E-06
**cholesteryl_esters_in_large_hdl**	0.607	0.2122	0.0042
**cholesteryl_esters_in_large_ldl**	-0.5767	0.1388	0
**cholesteryl_esters_in_large_vldl**	-4.9747	1.2546	1E-04
**cholesteryl_esters_in_medium_hdl**	0.0214	0.298	0.9429
**cholesteryl_esters_in_medium_ldl**	-1.7476	0.3398	2.7066E-07
**cholesteryl_esters_in_medium_vldl**	-2.0475	0.7458	0.006
**cholesteryl_esters_in_small_hdl**	-2.8779	0.6099	2.3733E-06
**cholesteryl_esters_in_small_ldl**	-4.172	0.8985	3.4269E-06
**cholesteryl_esters_in_small_vldl**	-3.0908	0.8627	3E-04
**cholesteryl_esters_in_vldl**	-0.7002	0.1994	4E-04
**cholesteryl_esters_in_very_large_hdl**	2.882	0.958	0.0026
**cholesteryl_esters_in_very_large_vldl**	-8.4317	2.0173	0
**cholesteryl_esters_in_very_small_vldl**	-1.4176	0.7931	0.0739
**cholesteryl_esters_to_total_lipids_in_chylomicrons_and_extremely_large_vldl_percentage**	-0.0083	0.0037	0.0247
**cholesteryl_esters_to_total_lipids_in_idl_percentage**	-0.0272	0.0121	0.024
**cholesteryl_esters_to_total_lipids_in_large_hdl_percentage**	0.0136	0.007	0.0514
**cholesteryl_esters_to_total_lipids_in_large_ldl_percentage**	-0.0811	0.0256	0.0015
**cholesteryl_esters_to_total_lipids_in_large_vldl_percentage**	0.0235	0.0083	0.0047
**cholesteryl_esters_to_total_lipids_in_medium_hdl_percentage**	0.0153	0.0119	0.1966
**cholesteryl_esters_to_total_lipids_in_medium_ldl_percentage**	-0.0498	0.0149	9E-04
**cholesteryl_esters_to_total_lipids_in_medium_vldl_percentage**	0.0099	0.0058	0.0887
**cholesteryl_esters_to_total_lipids_in_small_hdl_percentage**	-0.0283	0.0163	0.0815
**cholesteryl_esters_to_total_lipids_in_small_ldl_percentage**	-0.0472	0.0133	4E-04
**cholesteryl_esters_to_total_lipids_in_small_vldl_percentage**	2E-04	0.0106	0.9885
**cholesteryl_esters_to_total_lipids_in_very_large_hdl_percentage**	-0.0234	0.0107	0.0288
**cholesteryl_esters_to_total_lipids_in_very_large_vldl_percentage**	0.0074	0.0043	0.0839
**cholesteryl_esters_to_total_lipids_in_very_small_vldl_percentage**	-0.0018	0.0073	0.8092
**citrate**	-3.2958	2.2736	0.1472
**clinical_ldl_cholesterol**	-0.1653	0.0394	0
**concentration_of_chylomicrons_and_extremely_large_vldl_particles**	-45409.4535	20477.486	0.0266
**concentration_of_hdl_particles**	-21.7379	11.9537	0.069
**concentration_of_idl_particles**	-1117.1197	368.7925	0.0025
**concentration_of_ldl_particles**	-400.8223	102.9526	1E-04
**concentration_of_large_hdl_particles**	96.7105	36.1449	0.0075
**concentration_of_large_ldl_particles**	-577.0085	164.0951	4E-04
**concentration_of_large_vldl_particles**	-20873.7074	5525.4029	2E-04
**concentration_of_medium_hdl_particles**	-6.4778	31.503	0.8371
**concentration_of_medium_ldl_particles**	-1731.7673	387.5255	7.8666E-06

**concentration_of_medium_vldl_particles**	-10167.9183	2394.6942	0
**concentration_of_small_hdl_particles**	-110.1021	22.014	5.6897E-07
**concentration_of_small_ldl_particles**	-2981.4067	782.6399	1E-04
**concentration_of_small_vldl_particles**	-8125.3695	2282.7559	4E-04
**concentration_of_vldl_particles**	-2347.6642	658.3619	4E-04
**concentration_of_very_large_hdl_particles**	800.0132	278.9785	0.0041
**concentration_of_very_large_vldl_particles**	-46601.4422	13480.5429	5E-04
**concentration_of_very_small_vldl_particles**	-4373.7729	2057.0637	0.0335
**creatinine_nmr**	-0.7315	2.2247	0.7423
**degree_of_unsaturation**	-0.2858	0.3517	0.4165
**docosahexaenoic_acid**	-0.6083	0.33	0.0653
**docosahexaenoic_acid_to_total_fatty_acids_percentage**	-0.0076	0.0417	0.856
**free_cholesterol_in_chylomicrons_and_extremely_large_vldl**	-3.5579	1.5795	0.0243
**free_cholesterol_in_hdl**	0.3913	0.3852	0.3098
**free_cholesterol_in_idl**	-1.3195	0.5009	0.0084
**free_cholesterol_in_ldl**	-0.9378	0.2387	1E-04
**free_cholesterol_in_large_hdl**	2.0827	0.7329	0.0045
**free_cholesterol_in_large_ldl**	-1.3352	0.3698	3E-04
**free_cholesterol_in_large_vldl**	-4.9335	1.2193	1E-04
**free_cholesterol_in_medium_hdl**	0.0285	1.1738	0.9807
**free_cholesterol_in_medium_ldl**	-4.1563	0.9077	4.6693E-06
**free_cholesterol_in_medium_vldl**	-3.9082	0.9836	1E-04
**free_cholesterol_in_small_hdl**	-6.9388	1.7953	1E-04
**free_cholesterol_in_small_ldl**	-8.9174	2.3282	1E-04
**free_cholesterol_in_small_vldl**	-5.9349	1.5357	1E-04
**free_cholesterol_in_vldl**	-1.0523	0.2747	1E-04
**free_cholesterol_in_very_large_hdl**	11.4436	3.5827	0.0014
**free_cholesterol_in_very_large_vldl**	-7.2989	2.1163	6E-04
**free_cholesterol_in_very_small_vldl**	-3.6974	1.8999	0.0517
**free_cholesterol_to_total_lipids_in_chylomicrons_and_extremely_large_vldl_percentage**	0.0028	0.0065	0.6707
**free_cholesterol_to_total_lipids_in_idl_percentage**	-0.0193	0.0284	0.496
**free_cholesterol_to_total_lipids_in_large_hdl_percentage**	0.0502	0.0302	0.096
**free_cholesterol_to_total_lipids_in_large_ldl_percentage**	1E-04	0.0227	0.9973
**free_cholesterol_to_total_lipids_in_large_vldl_percentage**	-0.0116	0.0196	0.5527
**free_cholesterol_to_total_lipids_in_medium_hdl_percentage**	0.0597	0.0421	0.1566
**free_cholesterol_to_total_lipids_in_medium_ldl_percentage**	0.0092	0.0159	0.5631
**free_cholesterol_to_total_lipids_in_medium_vldl_percentage**	0.0163	0.0168	0.331
**free_cholesterol_to_total_lipids_in_small_hdl_percentage**	0.0783	0.0494	0.1128
**free_cholesterol_to_total_lipids_in_small_ldl_percentage**	-0.0089	0.0151	0.5538
**free_cholesterol_to_total_lipids_in_small_vldl_percentage**	-0.0015	0.0131	0.9081
**free_cholesterol_to_total_lipids_in_very_large_hdl_percentage**	-0.0315	0.0104	0.0025
**free_cholesterol_to_total_lipids_in_very_large_vldl_percentage**	0.0436	0.0153	0.0045
**free_cholesterol_to_total_lipids_in_very_small_vldl_percentage**	-0.0593	0.052	0.2537
**glucose_nmr**	0.0083	0.0261	0.7495
**glutamine**	0.123	0.3731	0.7416

**glycine**	0.8508	0.4291	0.0474
**glycoprotein_acetyls**	0.2047	0.2496	0.412
**hdl_cholesterol_nmr**	0.0901	0.0876	0.304
**histidine**	-9.7238	2.9692	0.0011
**isoleucine**	-4.7675	1.7326	0.0059
**ldl_cholesterol**	-0.2936	0.0664	9.6519E-06
**lactate**	-0.0418	0.027	0.1209
**leucine**	-4.9343	1.1121	9.1318E-06
**linoleic_acid**	-0.1085	0.0423	0.0103
**linoleic_acid_to_total_fatty_acids_percentage**	0.0075	0.0087	0.3832
**monounsaturated_fatty_acids**	-0.0805	0.037	0.0297
**monounsaturated_fatty_acids_to_total_fatty_acids_percentage**	-0.0016	0.0111	0.8893
**omega_3_fatty_acids**	-0.3154	0.1252	0.0118
**omega_3_fatty_acids_to_total_fatty_acids_percentage**	-0.0215	0.0177	0.223
**omega_6_fatty_acids**	-0.1366	0.0428	0.0014
**omega_6_fatty_acids_to_omega_3_fatty_acids_ratio**	0.022	0.0078	0.0047
**omega_6_fatty_acids_to_total_fatty_acids_percentage**	0.0103	0.0086	0.2309
**phenylalanine**	-3.0788	2.646	0.2446
**phosphatidylcholines**	-0.1428	0.0766	0.0622
**phosphoglycerides**	-0.1506	0.0726	0.0381
**phospholipids_in_chylomicrons_and_extremely_large_vldl**	-2.0958	0.9688	0.0305
**phospholipids_in_hdl**	0.0578	0.091	0.5257
**phospholipids_in_idl**	-1.0091	0.4245	0.0174
**phospholipids_in_ldl**	-0.9096	0.2065	0
**phospholipids_in_large_hdl**	0.5031	0.1851	0.0066
**phospholipids_in_large_ldl**	-1.4609	0.348	0
**phospholipids_in_large_vldl**	-2.8722	0.7681	2E-04
**phospholipids_in_medium_hdl**	-0.118	0.2931	0.6873
**phospholipids_in_medium_ldl**	-3.4812	0.7038	7.5663E-07
**phospholipids_in_medium_vldl**	-2.52	0.6138	0
**phospholipids_in_small_hdl**	-1.1578	0.3065	2E-04
**phospholipids_in_small_ldl**	-5.5502	1.573	4E-04
**phospholipids_in_small_vldl**	-3.73	0.9661	1E-04
**phospholipids_in_vldl**	-0.5935	0.1639	3E-04
**phospholipids_in_very_large_hdl**	2.1754	0.602	3E-04
**phospholipids_in_very_large_vldl**	-3.9716	1.1741	7E-04
**phospholipids_in_very_small_vldl**	-1.6284	1.0728	0.129
**phospholipids_to_total_lipids_in_chylomicrons_and_extremely_large_vldl_percentage**	-0.001	0.0066	0.8798
**phospholipids_to_total_lipids_in_idl_percentage**	0.0967	0.0321	0.0026
**phospholipids_to_total_lipids_in_large_hdl_percentage**	-0.0178	0.0108	0.0993
**phospholipids_to_total_lipids_in_large_ldl_percentage**	-0.0217	0.0383	0.5708
**phospholipids_to_total_lipids_in_large_vldl_percentage**	-0.0058	0.0076	0.4474
**phospholipids_to_total_lipids_in_medium_hdl_percentage**	-0.019	0.024	0.4298
**phospholipids_to_total_lipids_in_medium_ldl_percentage**	0.0392	0.0354	0.2675
**phospholipids_to_total_lipids_in_medium_vldl_percentage**	0.0141	0.0162	0.3839

**phospholipids_to_total_lipids_in_small_hdl_percentage**	0.0825	0.0248	9E-04
**phospholipids_to_total_lipids_in_small_ldl_percentage**	0.0596	0.0157	2E-04
**phospholipids_to_total_lipids_in_small_vldl_percentage**	0.0019	0.014	0.8912
**phospholipids_to_total_lipids_in_very_large_hdl_percentage**	0.0243	0.0059	0
**phospholipids_to_total_lipids_in_very_large_vldl_percentage**	0.0031	0.0104	0.7689
**phospholipids_to_total_lipids_in_very_small_vldl_percentage**	0.0836	0.034	0.014
**polyunsaturated_fatty_acids**	-0.1234	0.0361	6E-04
**polyunsaturated_fatty_acids_to_monounsaturated_fatty_acids_ratio**	0.043	0.0847	0.6113
**polyunsaturated_fatty_acids_to_total_fatty_acids_percentage**	0.0045	0.008	0.5722
**pyruvate**	-0.2292	0.7248	0.7518
**remnant_cholesterol**	-0.2488	0.0685	3E-04
**saturated_fatty_acids**	-0.0926	0.0319	0.0037
**saturated_fatty_acids_to_total_fatty_acids_percentage**	-0.0143	0.0157	0.3632
**sphingomyelins**	-0.7341	0.3973	0.0647
**total_cholesterol**	-0.0997	0.0303	0.001
**total_cholesterol_minus_hdl_c**	-0.1404	0.0342	0
**total_cholines**	-0.1404	0.0701	0.0451
**total_concentration_of_branched_chain_amino_acids**	-1.7803	0.3686	1.3638E-06
**total_concentration_of_lipoprotein_particles**	-26.6505	11.4401	0.0198
**total_esterified_cholesterol**	-0.1359	0.0421	0.0013
**total_fatty_acids**	-0.0385	0.0126	0.0022
**total_free_cholesterol**	-0.3594	0.1061	7E-04
**total_lipids_in_chylomicrons_and_extremely_large_vldl**	-0.3346	0.153	0.0288
**total_lipids_in_hdl**	0.0318	0.0449	0.4789
**total_lipids_in_idl**	-0.268	0.0974	0.0059
**total_lipids_in_ldl**	-0.2141	0.0486	0
**total_lipids_in_large_hdl**	0.2396	0.0863	0.0055
**total_lipids_in_large_ldl**	-0.3101	0.0763	0
**total_lipids_in_large_vldl**	-0.6905	0.174	1E-04
**total_lipids_in_lipoprotein_particles**	-0.0649	0.0176	2E-04
**total_lipids_in_medium_hdl**	-0.0407	0.1304	0.7548
**total_lipids_in_medium_ldl**	-0.9012	0.1794	5.0871E-07
**total_lipids_in_medium_vldl**	-0.6341	0.1446	0
**total_lipids_in_small_hdl**	-0.7808	0.1805	0
**total_lipids_in_small_ldl**	-1.9604	0.4546	0
**total_lipids_in_small_vldl**	-0.8334	0.2238	2E-04
**total_lipids_in_vldl**	-0.1292	0.0351	2E-04
**total_lipids_in_very_large_hdl**	1.122	0.3347	8E-04
**total_lipids_in_very_large_vldl**	-0.788	0.2294	6E-04
**total_lipids_in_very_small_vldl**	-0.5844	0.3186	0.0666
**total_phospholipids_in_lipoprotein_particles**	-0.1576	0.061	0.0098
**total_triglycerides_nmr**	-0.1652	0.0533	0.002
**triglycerides_in_chylomicrons_and_extremely_large_vldl**	-0.4805	0.2401	0.0454
**triglycerides_in_hdl**	-1.0883	0.608	0.0734
**triglycerides_in_idl**	-1.3729	1.1195	0.22
**triglycerides_in_ldl**	-1.5089	0.7322	0.0393

**triglycerides_in_large_hdl**	0.775	2.3349	0.74
**triglycerides_in_large_ldl**	-2.1222	1.1585	0.067
**triglycerides_in_large_vldl**	-1.3128	0.3383	1E-04
**triglycerides_in_medium_hdl**	-2.8625	1.4956	0.0556
**triglycerides_in_medium_ldl**	-6.9647	2.9493	0.0182
**triglycerides_in_medium_vldl**	-1.1131	0.2643	0
**triglycerides_in_small_hdl**	-4.799	1.6426	0.0035
**triglycerides_in_small_ldl**	-12.7609	5.432	0.0188
**triglycerides_in_small_vldl**	-1.4529	0.4927	0.0032
**triglycerides_in_vldl**	-0.2104	0.0641	0.001
**triglycerides_in_very_large_hdl**	-2.573	10.9259	0.8138
**triglycerides_in_very_large_vldl**	-1.2159	0.3807	0.0014
**triglycerides_in_very_small_vldl**	-1.995	1.3678	0.1447
**triglycerides_to_phosphoglycerides_ratio**	-0.3544	0.141	0.012
**triglycerides_to_total_lipids_in_chylomicrons_and_extremely_large_vldl_percentage**	0.0029	0.0023	0.1964
**triglycerides_to_total_lipids_in_idl_percentage**	0.0177	0.0123	0.1496
**triglycerides_to_total_lipids_in_large_hdl_percentage**	-0.0216	0.0109	0.0469
**triglycerides_to_total_lipids_in_large_ldl_percentage**	0.0435	0.0177	0.0142
**triglycerides_to_total_lipids_in_large_vldl_percentage**	-0.0044	0.0046	0.3353
**triglycerides_to_total_lipids_in_medium_hdl_percentage**	-0.0291	0.0163	0.0742
**triglycerides_to_total_lipids_in_medium_ldl_percentage**	0.0588	0.0195	0.0025
**triglycerides_to_total_lipids_in_medium_vldl_percentage**	-0.0049	0.0035	0.1586
**triglycerides_to_total_lipids_in_small_hdl_percentage**	-0.03	0.0224	0.1794
**triglycerides_to_total_lipids_in_small_ldl_percentage**	0.0176	0.0167	0.292
**triglycerides_to_total_lipids_in_small_vldl_percentage**	0	0.0044	0.9919
**triglycerides_to_total_lipids_in_very_large_hdl_percentage**	-0.0172	0.0091	0.0597
**triglycerides_to_total_lipids_in_very_large_vldl_percentage**	-0.0072	0.0034	0.0339
**triglycerides_to_total_lipids_in_very_small_vldl_percentage**	-9E-04	0.0074	0.8984
**tyrosine**	-7.2349	2.0353	4E-04
**vldl_cholesterol**	-0.4329	0.1171	2E-04
**valine**	-4.1388	0.7392	2.1572E-08
**neutrophill_ct_over_lymphocyte_ct**	0.1446	0.0121	7.9316E-33
**platelet_ct_over_lymphocyte_ct**	0.0012	2E-04	8.5269E-08
**asp_over_ala_aminotransferase**	0.3165	0.0338	7.7711E-21
**non_albumin_protein**	0.0028	0.004	0.4894
**eGFR**	6E-04	0.0154	0.9688
**Heel BMD**	-5.2377	0.1832	7.2089E-180

Table S2. The F statistic and their probabilities Pr(>F) values of traits determined in drop-one analysis. Significance thresholds are indicated by asterisks, where three asterisks indicate *p*-values below 0.001/14, two indicate p-values below 0.01/14, and one asterisk indicates *p*-values below 0.05/14.

Trait	F statistic	Pr(>F)
**Heel BMD**	116.0749	2.5901E-26
**Vitamin D**	26.2202	3.3544E-07
**Neutrophil count over lymphocyte count**	15.5852	1E-04
**Concentration of small HDL particles**	6.9742	0.0083

**Albumin**	6.0833	0.0137
**Mean sphered cell volume**	5.7844	0.0163
**Gamma glutamyltransferase**	5.0162	0.0252
**Urate**	2.2205	0.1364
**Erythrocyte count**	1.3253	0.2498
**High light scatter reticulocyte count**	1.2846	0.2572
**C-reactive protein**	1.0525	0.3051
**Asp over Ala aminotransferase**	1.0293	0.3104
**BMI**	1.0146	0.3139
**Lymphocyte count**	0.9478	0.3304
**SHBG**	0.5583	0.4551
**Sodium in urine**	0.3987	0.5278
**Mean platelet volume**	0.3771	0.5392
**Concentration of medium VLDL particles**	0.3452	0.5569
**Platelet count over lymphocyte count**	0.2547	0.6138
**IGF-1**	0.1503	0.6983
**Leucine**	0.0847	0.7711
**Cystatin C**	0.0802	0.7771
**Platelet distribution width**	0.0555	0.8138
**Potassium in urine**	0.0319	0.8583
**Platelet count**	0.0042	0.9482
**Erythrocyte distribution width**	0.0013	0.9711
